# Variation in resource allocation in urgent and emergency Care Systems in Ireland

**DOI:** 10.1186/s12913-019-4504-4

**Published:** 2019-09-11

**Authors:** Steve Thomas, Conor Foley, Bridget Kane, Bridget M. Johnston, Brenda Lynch, Susan Smith, Orla Healy, Elsa Droog, John Browne

**Affiliations:** 1 Centre for Health Policy and Management, Trinity College Dublin, 3-4 Foster Place, College Green, Dublin 2, Ireland; 2 Department of Epidemiology and Public Health, University College Cork, Cork, Ireland; 3 Department of General Practice, Royal College of Surgeons, Dublin, Ireland

**Keywords:** Emergency care, Health system, Resource allocation, Universal health care, Urgent care, Ireland

## Abstract

**Background:**

A key challenge for most systems is how to provide effective access to urgent and emergency care across rural and urban populations. Tensions about the placement and scope of hospital emergency services are longstanding in Irish political life and there has been recent reform to centralise hospital services in some regions. The focus of this paper is a system approach to examine the geographic variation in resourcing and utilisation of such care across GP practices, out-of-hours care, ambulance services, Emergency Departments and Local Injury Units in Ireland.

**Methods:**

We used a cross-sectional study design to evaluate variation in resource allocation by aggregating geographic funding to various elements of the urgent and emergency care system and assessing patterns in hospital resource utilisation across the population. Expenditure, staffing, access and activity data were gathered from government sources, individual facilities and service providers, health professional bodies, private firms and central statistics. Data on costs and activity in 2014 are collated and presented at both county and regional levels. Analyses focus on resources spent on urgent and emergency care across geographic areas, the role of population concentration in allocation, the relationship between pre-hospital spending and in-hospital spending, and the utilisation of hospital-based emergency care resources by residents of each county.

**Results:**

An array of funding mechanisms exists, resulting in a fragmented approach to the resourcing of urgent and emergency care. There are large differences in spending per capita at the county-level, ranging from between €50 and €200 per capita; however, these are less pronounced regionally. Distribution of hospital emergency care resources is highly skewed to the North East of the country, and away from the recently reconfigured South and Mid-West regions.

**Conclusions:**

This analysis advances the traditional approach of evaluating individual services or hospital resourcing. There are notable differences in utilisation of hospital-based emergency care resources at the regional level, indicating that populations within those regions which have been reconfigured have lower utilisation of hospital resources. There is a clear case for more integration in decision-making around funding and consideration of key principles, such as equity, to guide that process.

**Electronic supplementary material:**

The online version of this article (10.1186/s12913-019-4504-4) contains supplementary material, which is available to authorized users.

## Introduction

Many international studies have highlighted geographical variation in survival from emergency conditions [1–6]. Rural areas are associated with a higher risk for poor outcomes from emergency conditions for a number of reasons including older and more socioeconomically disadvantaged populations, longer travel times to tertiary treatment centres and concerns about the quality of care delivered at smaller rural hospitals [2, 6, 7]. Measures to address this problem are controversial. One approach is to concentrate services in a smaller number of specialist services. In theory, this transports patients directly to a setting that is appropriate to the severity of their condition, but also exacerbates the underlying risk associated with rurality by lengthening journey times [8].

Emergency care services have been reconfigured across the Republic of Ireland since 2006, with notable regional variation in planning and implementation [9–12]. Reform initiatives, where implemented, have focused on directing patients to settings that are appropriate for their care needs. These programmes typically featured reduced access to emergency care in local hospitals, centralisation of specialist emergency services in ‘hub’ hospitals, and development of integrated, condition-specific referral protocols for both ambulance and general practice services. The majority of service reconfiguration was carried out in the southern and western regions of the country, which are also the most sparsely populated areas of the country.

**Fig. 1:**
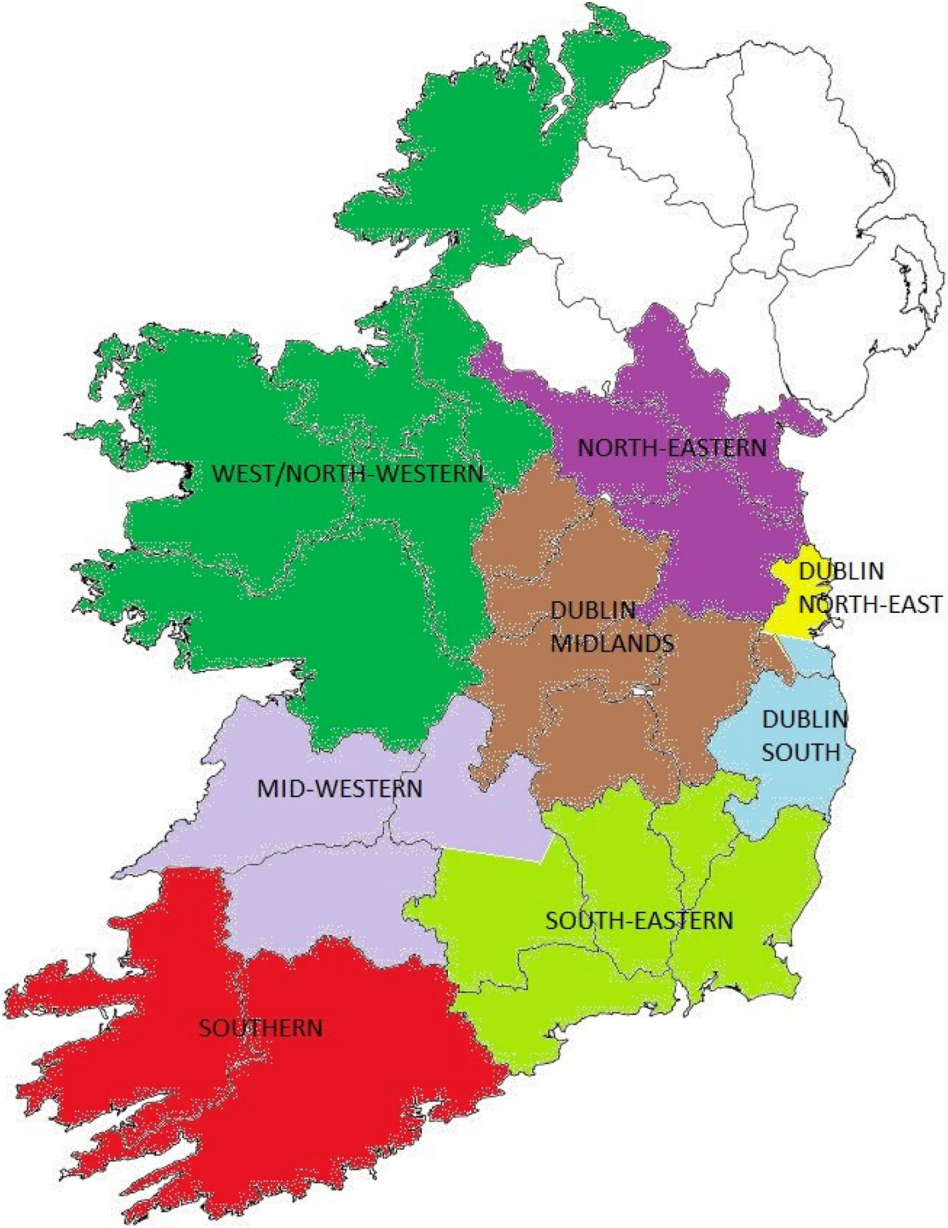
Operational regions of Ireland (2014)

The Republic of Ireland is an island of 70·2 thousand km^2^ with a population of 4·5 million, divided into 26 counties. At the time of the project design, there were eight operational regions in the Irish health care system (Fig. 1). The characteristics of each region and measures taken to reconfigure services are presented in Table 1. Two regions (South and Mid-West) have implemented significant reconfiguration of urgent and emergency care [9, 11]. Four regions (West, North-East, Dublin South, and South-East) have introduced some measures designed to reconfigure care but these do not cover all services [9–11]. Two regions (Dublin Midlands and Dublin North-East) have undertaken no major changes to the configuration of urgent and emergency care since 2006 [9–12].

**Table 1:** Reconfiguration of emergency care systems in Ireland

Region	Characteristics^a^	Summary of regional changes
North East (Cavan, Meath, Louth and Monaghan)	Population: 440,211 Area (km^2^): 6395	• Region-specific reconfiguration plan partly implemented from 2006 to 2010. • Two emergency departments reconfigured to local injury units. • Some centralisation of trauma, acute stroke and coronary care (to Cavan and Louth) with rehab support in other hospitals. • Mater [Dublin North] is the percutaneous coronary intervention (PCI) centre with supporting ambulance protocols. • Roll-out of general practitioner (GP) out of hours care. • Limited regional clinical governance.
Dublin North East (Fingal, Dublin North City)	Population: 578,317 Area (km^2^): 532	• No major changes. • Three large emergency departments with limited governance integration and differentiation of services (PCI centre at Mater [Dublin North]). • Out of hours GP co-operative established.
Dublin South (Dublin South City, Dun Laoghaire Rathdown, Wicklow)	Population: 563,560 Area (km^2^): 2168	• One emergency department reconfigured to local injury unit in 2013, with reduced hours in another. • Centralisation of acute stroke, coronary and trauma care to two hospitals (both in Dublin South City) but limited differentiation and integration between both. • Multiple out of hours GP co-operatives.
Dublin Midlands (Dublin South, Longford, Westmeath, Laois, Offaly, Kildare)	Population: 761,324 Area (km^2^): 8442	• Centralisation of acute stroke (Kildare, Westmeath, and Dublin South) coronary care (Dublin South) and trauma (Offaly and Dublin South) at several hospitals, supported by ambulance bypass protocols. • Limited integration of clinical governance. • Several out of hours GP co-operatives operating.
South East (Carlow, Kilkenny, Wexford, Waterford and Tipperary South)	Population: 497,305 Area (km^2^): 9451	• Designated hub for major trauma, and acute coronary care (Waterford – PCI centre supported out of hours by Cork) with ambulance bypass protocols. • Acute stroke care available at all 4 hospitals. • Informal clinical network with shared regional rota for emergency medicine consultants. • Single GP out of hours co-operative.
South (Cork and Kerry)	Population: 663,176 Area (km^2^): 12,161	• Region-specific reconfiguration plan largely implemented, beginning 2012–2013. • Two emergency departments reconfigured to local injury units, with another closing. • Acute stroke, coronary and major trauma care provided at hub CUH [Cork] with support of ambulance protocols and outlying centres (Kerry can thrombolyse and deal with most trauma and myocardial infarctions (MIs), Bantry [Cork] does thrombolysis). • Region-wide clinical governance structures established. • Single GP out of hours co-operative.
Mid-West (Limerick, Clare and Tipperary North)	Population: 378,210 Area (km^2^): 8252	• Region-specific reconfiguration plan largely implemented, 2009–2013. • All emergency care centralised to one hospital (Limerick), former emergency departments reconfigured to local injury units. • Ambulance bypass protocols and region-wide clinical directorates established. • Single GP out of hours co-operative.
West (Galway, Roscommon, Mayo, Leitrim, Sligo, Donegal)	Population: 702,966 Area (km^2^): 22,649	• Reconfiguration of one emergency department to a local injury unit in 2011. • Single hub for acute coronary (Galway is the PCI centre but Sligo and Roscommon have a mobile cath. Lab 1 day a week) and major trauma care with support services provided at other centres (Mayo, Donegal and Sligo take most trauma cases). • Acute stroke care available at all centres, excluding Roscommon. • Clinical directorates established across the region. • Several out of hours GP co-operatives.

^a^ Source: Central Statistics Office [13]

It is unclear whether the composition of regional funding for emergency care has responded to these initiatives. Populations in some areas now have to travel much further to access an emergency department, and this should imply a commensurate increase in pre-hospital resources such as out-of-hours general practice and ambulance services. However, the reconfiguration of services coincided with a deep financial crisis for Ireland’s public health system: it has been estimated that public funding for health was reduced by 22% over the period 2009 to 2013 [14]. This crisis may have made it difficult for policy makers to properly match emergency care resources to population need; thus, it is possible that both horizontal and vertical inequities have arisen or been exacerbated across geographical areas in Ireland. By horizontal inequities, we mean that urgent and emergency care systems may not provide the same services for people with similar needs, regardless of where they come from geographically. Vertical inequities arise when people with worse economic circumstances do not get better access.

The aim of this research is to examine variation in resource allocation to urgent and emergency care systems and utilisation of such resources across Ireland. This is evaluated by comparing overall resource levels across counties and analysing county-level resource composition in relation to population need. The key research questions are:

1. Are resources for urgent and emergency care distributed evenly across counties and regions?
2. Are those areas with lower population density or deprivation associated with higher per capita funding levels?
3. Do regions which have reconfigured have higher non-hospital spending to compensate for the centralisation of services?
4. Is there equal utilisation of hospital-based emergency care resources across counties and regions, regardless of configuration?

## Methods

A cross-sectional study design was used to collect data on resourcing and utilisation in urgent and emergency care in Ireland. This comprises services provided in either the community (pre-hospital) or in hospitals (in-hospital). Pre-hospital expenditure includes spending on GP and practice nurse services (in and out of hours) and ambulance services. In-hospital expenditure includes spending on Emergency Departments (ED) and Local Injury Units (LIU) in public and private hospitals. Data on activity, staffing and expenditure were gathered from a range of sources, including government agencies, individual facilities and service providers, health professional bodies and private firms, and the Health Service Executive (HSE) (Table 2). Information on county and regional populations, as well as geographical area, were taken from the most recent census information provided by the Central Statistics Office [13].

**Table 2:** Data sources

Service	Details of data collected
Pre-hospital Expenditure	
General Practice: Activity, Funding and Staffing	• Claims through the Primary Care Reimbursement Service (PCRS) categorised as ‘Emergency’ and ‘Out of Hours’ were taken to represent urgent and emergency cases [15]. Expenditure related to these claims is also reported by the PCRS. • GP and practice nursing workforce figures were reported in a recent study [16]. • Data on workforce employed by GP co-operatives were gathered through surveys sent to the General Manager of each practice (response rate = 90%). • Additional data on healthcare service utilisation taken from the Quarterly National Household Survey [17] were used to estimate GP and practice nurse staffing and non-medical card activity and expenditure
Ambulance Services: Activity, Funding and Staffing	• Data on ambulance staffing was provided by the National Ambulance Service [18, 19], Dublin Fire Brigade, the Health Service Executive and Health Information and Quality Authority [20].
In-Hospital Expenditure	
Public ED and LIU: Activity, Funding and Staffing	• Public ED and LIU expenditure data were provided by the Health Service Executive’s (HSE) Healthcare Pricing Office (HPO). • Overall Hospital expenditure data were extracted from the HSE Management Data report [21]. • Surveys designed to collect additional data on ED and LIU consultant, non-consultant hospital doctor and nursing staffing levels were posted to all hospitals (Response rate = 33%). Secondary sources were used to supplement the survey data [22, 23]. • Presentations at each public ED and LIU were extracted from the HSE Data Management report [21].
Private ED and LIU: Activity, Funding and Staffing	• Data on consultant staffing and activity in private EDs were estimated by the HSE, based on contractual agreements with consultants around the split between public and private work. • Data on the number of presentations at private LIUs were used to estimate resources used in private clinics [22].
County of Residence: Utilisation	• The patient’s county of residence in relation to each ED and LIU presentation was extracted from the Hospital In-Patient Enquiry Scheme, held by the HPO.
Population Estimates	
County and Regional	• All population data were taken from the 2011 Census [13].

Ethical approval was granted by the Clinical Research Ethics Committee of the Cork Teaching Hospitals prior to study commencement.

### Pre-hospital expenditure

Several datasets were used in compiling and estimating figures on staffing, activity and expenditure in general practice, both in and out of hours. Activity and expenditure figures for the General Medical Scheme (GMS) and GP Visit Card Scheme (GPVC) primary care patients were available from the Health Service Executive’s (HSE) Primary Care Reimbursement Service (PCRS) [15] and they cover around 40% of the population. These patients are entitled to free GP care, based on means-testing and some health condition-related circumstances. Under the PCRS system, claims are broken down into several categories. For the purposes of the current study, claims categorised as ‘Emergency’ and ‘Out of Hours’ were taken to represent urgent and emergency cases.

PCRS figures are reported according to Local Health Office (LHO) boundaries which are not coterminous with county boundaries. As a result, figures for individual counties were estimated based on population for LHOs containing two counties and computed for counties with multiple constituent LHOs. Regional figures were compiled by summing the values for their constituent LHOs. In some areas such as Dublin, LHO figures were split according to the proportion of the population living in each region.

There is no precise figure for the GP workforce by county due to the lack of a single, central register. A recent study [16] estimated the number of GPs and practice nurses by combining data from three sources: the 2010 GMS payments list; the Irish College of General Practitioners’ ‘Find a GP’ service (extracted January 2011); and the 2010/2011 Irish Medical Directory. These figures for GPs and practice nurses were used in the current study.

Staff numbers for out of hours GP co-operatives were gathered through a survey conducted with the assistance of the PCRS in 2014. This survey gathered data on whole-time equivalents (WTE) for administrative, nursing and support staff employed by co-operatives. These were posted to the General Manager of each of the ten co-operatives identified and nine completed and returned the survey, giving a response rate of 90%.

GP and practice nurse staffing specifically for urgent and emergency care was estimated based on PCRS activity figures for emergency and out of hours medical card claims as a proportion of total medical card claims for 2013. Quarterly National Household Survey (QNHS) [17] data on healthcare service utilisation were used to estimate staffing for non-medical card urgent and emergency care. These data suggested that medical card patients were 2.44 times more likely to attend their GP than non-medical card patients. By calculating the proportion of non-medical card to medical card eligible populations in each county and taking into account the QNHS figure it was possible to estimate non-medical card primary care emergency and out of hours activity, and staff WTE devoted to it. Non-medical card activity and expenditure were estimated on the same basis, with an assumption that each non-medical card GP visit cost the patient €60 [24].

Information on ambulance staffing, activity and funding was provided by the National Ambulance Service (NAS), Dublin Fire Brigade (DFB), HSE and also extracted from the Health Information and Quality Authority (HIQA) review of ambulance care [18–20]. Ambulance service staffing by county (with the exception of the majority of County Dublin, which is served by the DFB) was calculated based on figures provided by NAS detailing the number of staff employed at each ambulance base in 2015. For Dublin, DFB provided total staffing figures, which included fire and ambulance personnel, taken from a 2013 internal audit and subsequent review in 2015. The number of operational ambulance personnel was estimated based on the proportion of funding for DFB ambulance activity (approx. €18 m based on HIQA report figure), compared to DFB’s total funding of approximately €111 m. Ambulance funding by county was estimated by dividing the total NAS budget proportionately according to staffing WTE per county.^1^ Regional figures were calculated in a similar manner.

### In-hospital expenditure

Public ED and LIU expenditure figures for each hospital were provided by the HSE’s Healthcare Pricing Office (HPO), while overall expenditure per hospital was sourced from the HSE Management Data Report [21]. ED and LIU expenditure in 2014 was estimated by taking the proportion of ED/LIU expenditure to total expenditure on 2013 and applying it to the total expenditure figures for 2014.

Establishing precise figures for ED and LIU consultant, non-consultant hospital doctor and nursing staff required amalgamating data from secondary sources [22, 23] to supplement new information requested through a postal survey from each hospital (response rate = 33%). Data on consultant staffing at private EDs were estimated by the HSE. These were used to estimate private hospital resources in emergency care. Data on the number of presentations at private LIUs were used to estimate resources consumed in the three private clinics identified in Dublin and Galway.

Public ED and LIU figures for the number of presentations at each hospital were available through the HSE Management Data Report [21]. Utilisation data for each hospital ED or LIU in relation to a patient’s county of residence was gained from the Hospital In-Patient Enquiry Scheme, held by the HPO. This allowed for analysis of the utilisation of hospital-based emergency care by residents of each county. Furthermore by calculating the average cost of presentation at each hospital and the county of residence for each patient, the authors estimate and compare the resources utilised across each county. In this case some hospitals primarily serve the residents of other counties and so resources are then allocated to the county of patient residence and not to the county where the hospital is situated.

### Analysis

All analyses were conducted using Microsoft Excel. Total costs for pre-hospital and in-hospital spending were calculated for each region and divided across the population to derive per capita costs for each region. The association between population density and per capita spending on pre-hospital care was examined using a scatterplot and linear trendline.

A key problem with evaluating resources spent in each county per capita to determine utilisation is that many residents will travel to other counties to access services. In some cases there are no or fewer local services, and in other cases specialist services are required that are only available from a few or even one facility in the country. Furthermore, cross border care from in the UK is free and so patients near the border do not face cost-barriers when gaining access there.

One way of addressing this is to consider the distribution of resources according to key regions. The logic of this is that some counties will group together to deliver care. For instance, Cork and Kerry have strong linkages as do Kildare, Meath and Dublin. Additionally, it is also useful to estimate the total resources spent on the urgent and emergency care of each resident of a county by determining their utilisation of these services in facilities across all counties.

## Results

### Are urgent and emergency care resources distributed evenly across counties and regions?

A breakdown of the overall financing per capita of urgent and emergency care services by each county in 2014 in Euros is shown in Table 3. This also disaggregates spending by resources flowing through General Practice, Ambulance services and public hospital EDs and LIUs.

**Table 3:** Spending per capita on Urgent and Emergency Care by county and region, Euro 2014

Region/County	GPs	Ambulance	Public EDs and LIUs	Total
North East				
Cavan	1.94	39.97	123.89	165.80
Louth	7.35	47.96	152.37	207.68
Meath	16.85	17.99	33.32	68.16
Monaghan	1.95	55.27	13.37	70.59
Dublin North East				
Dublin	13.26	22.45	109.93	145.64
Dublin South				
Dublin	13.26	22.45	109.93	145.64
Wicklow	9.65	37.00	0.00	46.65
Dublin Midlands				
Dublin	13.26	22.45	109.93	145.64
Kildare	14.54	21.85	32.92	69.32
Laois	13.19	33.25	87.71	134.14
Longford	18.26	59.35	0.00	77.62
Offaly	13.19	61.75	100.30	175.24
Westmeath	18.27	55.96	65.15	139.38
South East				
Carlow	39.17	38.25	0.00	77.42
Kilkenny	39.17	29.19	52.65	121.01
Tipperary	13.53	59.05	60.83	133.93
Waterford	10.62	41.61	96.70	148.93
Wexford	12.08	32.59	55.05	99.71
South				
Cork	22.62	32.86	74.24	129.72
Kerry	26.82	58.66	55.32	140.80
Mid-West				
Clare	12.63	58.85	14.31	85.79
Limerick	21.19	26.14	100.83	148.16
Tipperary	13.53	59.05	60.83	133.93
West				
Donegal	14.31	54.94	52.87	122.11
Galway	14.29	36.91	90.24	141.43
Leitrim	10.10	64.82	0.00	74.93
Mayo	16.76	49.68	40.17	105.65
Roscommon	10.44	71.52	28.24	110.21
Sligo	10.10	25.34	129.52	164.97
National Average	*15.66*	*31.47*	*81.28*	*131.06*

The county of Dublin spans three regions (Dublin North-East, Dublin South and Dublin Midlands), while Tipperary spans two regions (Mid-West and South-East). Consequently Dublin and Tipperary appear more than once in the table

As seen, there is substantial variation in urgent and emergency care funding across counties – ranging from over €200 per capita in Louth, to under €50 per capita in Wicklow. There are also notable differences in patterns of funding for the various components of urgent and emergency care. For example, funding for GPs by county varies from under €2 per capita in Cavan and Monaghan to almost €40 per capita in Kilkenny and Carlow. Additionally, spending on ambulance services ranges from €17 in Meath to over €70 per capita in Roscommon, while for EDs and LIUs it varies from €0 in Leitrim to €152 in Louth.

### Are those areas with lower population density or deprivation associated with higher per capita funding levels**?**

We examined the association between population density and deprivation and funding of pre-hospital urgent and emergency care (Dublin is excluded as the population density is extremely high compared to the other counties). The data are inconclusive for population density and pre-hospital funding per capita in general; however, there are significant outliers^2^ (Additional file 1: Figure S1). There appears to be no link between pre-hospital funding and deprivation (Additional file 2: Figure S2).

With regard to the resourcing of ambulance services, many rural counties on the Western seaboard such as Donegal, Clare, Kerry, Roscommon and Leitrim are relatively well resourced (Table 2). However, the pattern is not consistent as Mayo and Cork, also with dispersed rural populations, are not well served. Instead, the highest level of resource per capita funding appears to be in the Midland counties of Longford, Offaly and Westmeath. Part of this may be understood in that specialist emergency care may only be provided in other counties (e.g., Dublin, Cork or Galway). Nevertheless, the resourcing of ambulance services across counties would seem to be inconsistent and unexplained by rurality.

### Do those regions which have reconfigured have higher non-hospital spending to compensate for the centralisation of services?

The spending of resources on urgent and emergency care across HSE regions is presented in Table 4. It is interesting how similar overall per capita funding levels are, though the division of such funds shows remarkably different models of delivering care across the regions. There is a concentration of hospital resources per capita in the North East, Dublin North East and Dublin South, combined with low pre-hospital spending, particularly in Dublin North East. Furthermore, private emergency care services are concentrated to parts of Dublin and Cork and is responsible for much of the variation. There is some evidence to suggest that those regions which have had reconfiguration have higher pre-hospital expenditure, with the South, the West and the Mid-West regions having the highest levels of such spending.

**Table 4:** Regional spending per capita in urgent and emergency care, 2014 Euro

	Pre-hospital Expenditure	Public ED and LIU Expenditure	Private ED and LIU Expenditure	Overall Expenditure
Dublin South	46	79	14	139
Dublin North East	33	98	7	138
South	62	61	9	132
West	61	61	6	127
North East	45	79	0	124
South East	59	64	0	123
Mid West	60	60	0	120
Dublin Midlands	42	63	0	105

Pre-hospital expenditure includes spending on GP and practice nurse services (in and out of hours) and ambulance services

### Is there equal utilisation of emergency care resources in hospitals across counties and regions regardless of configuration?

**Fig. 2:**
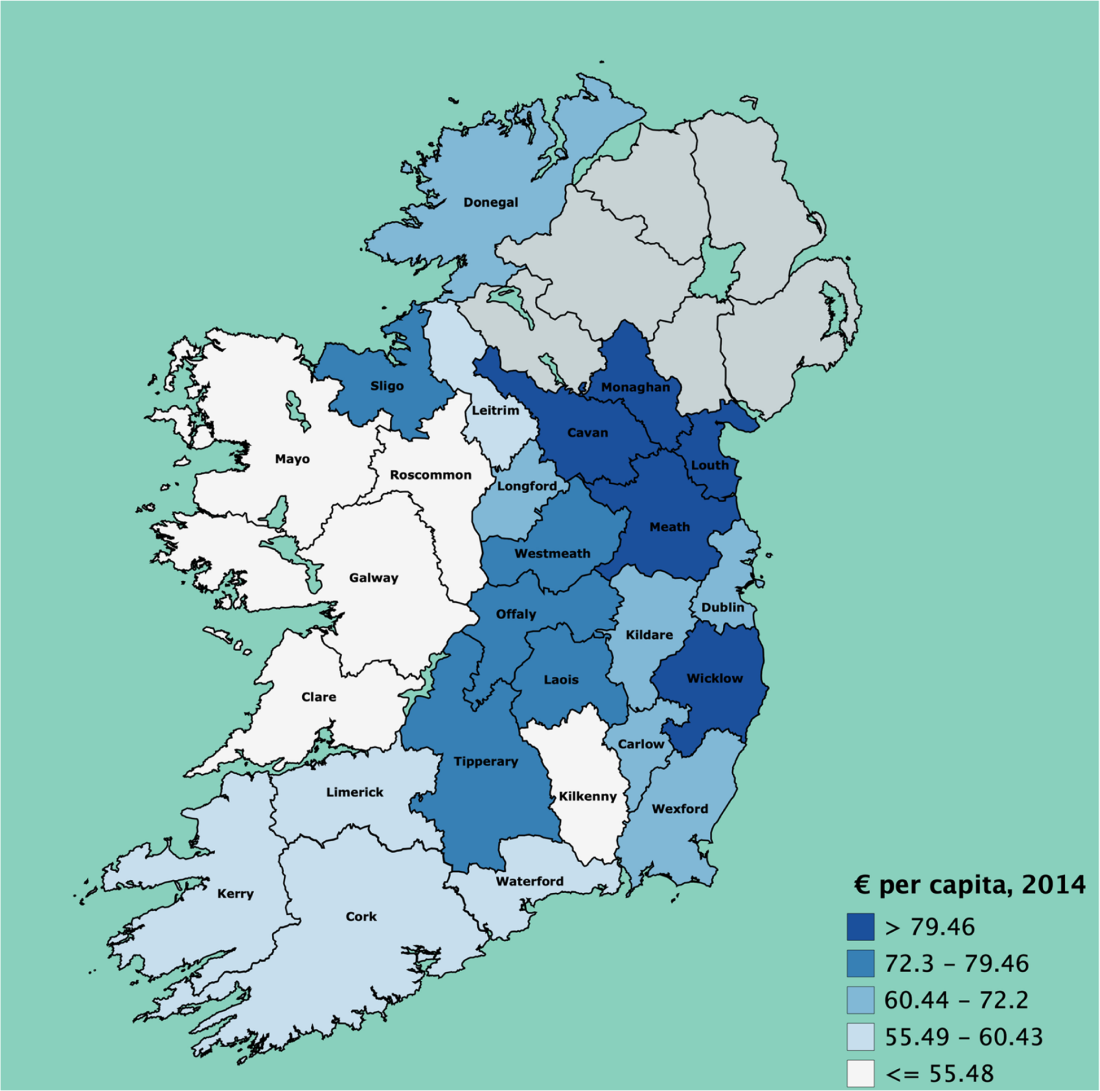
Hospital (ED and LIU) resources utilised by residents of each county (€ per capita 2014)

To capture the hospital emergency care resources utilised across counties, the authors allocate resources from local hospitals back to the county of residence of each patient. Figure 2 provides a picture of this allocation across patient county of residence. It appears there are profound imbalances across the country in utilisation of hospital resources for urgent and emergency care. For example, there is much higher consumption of hospital resources by residents in the North East of the country, with four of the top five counties being from this region. Additionally, residents in the Midlands have higher utilisation of hospital resources – partly because of ease of access to hospitals Dublin and Galway, but also attributed to high local hospital spending.

Conversely, many counties in the West and the South do relatively poorly in relation to their residents utilising hospital resources for urgent and emergency care. Utilisation in the West, bar Sligo and Donegal, seems particularly poor. Further, the low per capita spending on residents of Kilkenny may be because some emergency care is conducted outside of EDs in the local hospital.

## Discussion

The results highlight wide disparities in the funding of care across counties, and to a lesser extent regions, with quite different models of care and unequal utilisation of hospital resources. It is interesting, and perhaps unexpected, to see that Louth with €207 per capita has the highest per capita spending for Urgent and Emergency Care followed by Offaly, Cavan and Sligo. These are all small counties with small populations and yet with significant hospital spending on emergency care. These results more than likely reflect the fact that hospitals located in counties with lower population density have a smaller base to spread fixed costs over than those in more densely populated counties. Overall, those counties with the lowest spending per capita either have no local hospital or are largely reliant on ED and/or LIU services from neighbouring counties.

It is not surprising that more rural counties have higher spending on pre-hospital care, particularly where there is no local hospital or where the population is dispersed and travel times to facilities are often substantial. In such cases ambulance costs will be higher and perhaps GP costs. Nevertheless, there is no consistency of funding in relation to rurality, particularly for ambulance care.

A key question is whether any of the variation identified have been the result of deliberate health care policy and a systemic approach to planning urgent and emergency care. Certainly 2014 marked the end of a prolonged austerity period for the Irish health care system with substantial pressure on government to cut public resources for health care [13].

The picture that emerges though in relation to funding urgent and emergency care is one of fragmentation. Hospital budgets have no specific cost centre for emergency care and there has been no mechanism for activity based funding for EDs. Instead funding for emergency departments comes from general funds through budgeting which is both institutional and incremental. This means that history and past politics have been important to the development of emergency care in hospitals. Nevertheless, it is hoped that activity based funding will be introduced at some stage in this area. Although activity based funding is commonly used to fund acute hospital care, there is not a large body of research about the impact of this funding mechanism on efficiency, particularly in emergency and urgent care [25], and the findings vary between countries and contexts [26] . Further research on costs, cost-effectiveness and the implications of various staff-mix configurations are needed to ensure that activity based funding captures the complexity of cases treated in ED and urgent care settings appropriately [25]. There may also be potential for more integration of services through the relatively new hospital networks. Ambulance services are funded separately for National Ambulance Services (outside of Dublin) and the Dublin Fire Brigade (within Dublin). GPs are entirely private sector agents but are funded by Government through a contract for those patients who have medical cards. Their contract is based on a mix of per capita payments and fee for services [27]. The out of hours and emergency care that they provide for medical card patients is funded on a fee for service basis by Government. Private patients (around 60% of the population) who wish to receive such care, must pay the full costs. Individuals who wish to seek private emergency care may do so in the major cities, according to their own means and private insurance status.

This array of funding mechanisms has resulted in a piece-meal approach to the resourcing of urgent and emergency care. There is a clear case for more integration in decision-making around funding, and consideration of key principles, such as equity, to guide that process. Only then might there be scope for moving toward a system appropriately resourced for universal access to quality emergency care.

### Limitations

Choosing the appropriate geographic unit of analysis is not straight forward when some counties have a full range of facilities and other neighbouring counties do not. In some cases the region is a more natural locus of investigation as in the West or the South of the country. In other cases the movement of patients is more fluid beyond regional boundaries. The analysis takes note of appropriate idiosyncrasies.

It is important to capture all of the resources in relation to urgent and emergency care [28]. This is not easy and the study has incorporated primary, ambulance and hospital care services. However, limitations may relate to some specialised units in hospitals which have in effect undertaken emergency care which are not captured here. Also there may be some primary care providers, such as community nurses, which also may initiate urgent care. Nevertheless, the analysis advances the traditional approach of evaluating individual services or hospital resourcing by instead focusing on the entire system.

## Conclusions

The approach taken to evaluate variation in resource allocation in urgent and emergency care systems is unique by aggregating geographic funding to different elements of the system and evaluating resource utilisation by different populations. The results show inequities in overall resource allocation by county, and to some extent, by region. There are also strong inequities in utilisation of resources provided for emergency hospital services between residents in the North East region of the country and those in the South and West. The results showed some resource compensation in counties and regions with few or centralised hospital services with higher pre-hospital funding. However, such higher funding appears to only partially compensate for utilisation of effective specialised care especially in relation to those regions that have been reconfigured. There is need for a more integrated resource allocation process to ensure appropriate and more equitable resourcing across the different elements of the system.

## Additional files

Additional file 1: **Figure S1** Population density and pre-hospital funding per capita across counties. Scatterplot examining patterns in pre-hospital funding per capita and population density across counties. (DOCX 18 kb)

Additional file 2: **Figure S2.** Deprivation index and pre-hospital funding per capita across counties. Scatterplot examining patterns in pre-hospital funding and deprivation across counties. (DOCX 153 kb)

## Data Availability

The datasets used and/or analysed during the current study are available from the corresponding author on reasonable request.

## Abbreviations

DFB: Dublin Fire Brigade
ED: Emergency Departments
GMS: General Medical Scheme
GP: General Practitioner
GPVC: GP Visit Card
HIQA: Health Information and Quality Authority
HPO: Healthcare Pricing Office
HSE: Health Service Executive
LHO: Local Health Office
LIU: Local Injury Unit
MI: Myocardial infarction
NAS: National Ambulance Service
PCI: Percutaneous coronary intervention
PCRS: Primary Care Reimbursement Service
QNHS: Quarterly National Household Survey
WTE: Whole Time Equivalent

## Footnotes

1 This includes the air ambulance service.

2 Outlier counties are Carlow, where spending is relatively high on GPs but where there is also quite high population density; Louth which has very high population density and intermediate levels of pre-hospital spending and Sligo which has both low population density and low pre-hospital funding but where there is a substantial hospital funding (Cavan and Laois display similar features).
