# Supplementary material for: Variation in resource allocation in urgent and emergency Care Systems in Ireland
Source: BMC Health Serv Res. 2019 Sep 11;19:657. doi: 10.1186/s12913-019-4504-4 (PMC6737720; doi:10.1186/s12913-019-4504-4)
Supplement: Supplementary file 1 — Figure S1 Population density and pre-hospital funding per capita across counties. Scatterplot examining patterns in pre-hospital funding per capita and population density across counties. (DOCX 18 kb) [file 12913_2019_4504_MOESM1_ESM.docx]

**Figure S1. Population density and pre-hospital funding per capita across counties**
