# Supplementary material for: Variation in resource allocation in urgent and emergency Care Systems in Ireland
Source: BMC Health Serv Res. 2019 Sep 11;19:657. doi: 10.1186/s12913-019-4504-4 (PMC6737720; doi:10.1186/s12913-019-4504-4)
Supplement: Supplementary file 2 — Figure S2. Deprivation index and pre-hospital funding per capita across counties. Scatterplot examining patterns in pre-hospital funding and deprivation across counties. (DOCX 153 kb) [file 12913_2019_4504_MOESM2_ESM.docx]

**Figure S2. Deprivation index and pre-hospital funding per capita across counties**
